# Preparation and High-Sensitivity Thermochromic Performance of MXene-Enhanced Cholesteric Liquid Crystal Microcapsule Textiles

**DOI:** 10.3390/polym18020223

**Published:** 2026-01-15

**Authors:** Xuzhi Sun, Yi Yang, Xiangwu Zhang, Maoli Yin, Mingfei Sheng

**Affiliations:** 1 School of Textile and Garment, Anhui Polytechnic University, Wuhu 241000, China; 657174979@mail.ahpu.edu.cn (X.S.); 15961781616@163.com (Y.Y.); 2 Key Laboratory of Clean Dyeing and Finishing Technology of Zhejiang Province, Shaoxing University, Shaoxing 312000, China; 3 Xuancheng Kaiou New Material Co., Ltd., Xuancheng 242000, China; 18956308568@163.com

**Keywords:** MXene, cholesteric liquid crystal, microcapsules, thermochromism

## Abstract

To mitigate the attenuation of color-change sensitivity in cholesteric liquid crystals (CLCs) post-microencapsulation, this study developed MXene-reinforced thermochromic textiles. Monolayer/few-layer MXene nanosheets were fabricated via an etching-intercalation-dispersion approach, while cholesteric liquid crystal microcapsules (CLCMs) were synthesized through a solvent evaporation method. Cotton fabrics were pretreated with polydopamine (PDA), followed by the fabrication of poly(diallyldimethylammonium chloride) (PDAC)/MXene composite coatings via layer-by-layer (LbL) self-assembly and subsequent hydrophobic modification. Systematic characterizations (scanning electron microscopy, SEM; atomic force microscopy, AFM) and performance evaluations revealed that MXene nanosheets have an average thickness of 1.54 nm, while CLCMs display a uniform spherical morphology. The resultant textiles exhibit a reversible red-green-blue color transition over the temperature range of 26.5–29.5 °C, with sensitivity comparable to pristine CLCs and excellent hydrophobicity. This work overcomes the long-standing bottleneck of inadequate color-change sensitivity in conventional liquid crystal microcapsule textiles, offering a novel strategy for the advancement of smart wearable color-changing materials.

## 1. Introduction

Wearable smart color-changing textiles have emerged as a frontier research direction in smart materials and textile engineering, driven by their irreplaceable application prospects in healthcare monitoring, the Internet of Things (IoT), and wearable biosensing [1,2,3]. As a core category of thermochromic materials, cholesteric liquid crystals (CLCs) distinguish themselves with ultrahigh temperature sensitivity (detection limit: 0.1 °C) and rapid response kinetics—properties that have enabled their practical deployment in non-destructive testing, heat flux sensing, and clinical temperature monitoring [4,5,6]. These unique characteristics render CLCs ideal functional carriers for developing high-performance smart color-changing textiles, effectively addressing the growing demand for precise and responsive wearable sensing platforms [7,8,9].

Current fabrication strategies for color-changing textiles primarily fall into two categories: one involves internal doping of color-changing agents into fibers, followed by spinning and weaving to form integrated textiles, while the other relies on surface immobilization of color-changing materials onto fabrics or within fiber gaps via textile finishing technologies [10,11,12]. For CLCs—characterized by inherent fluidity, poor fixability, and sensitivity to environmental factors (e.g., humidity, mechanical friction)—microencapsulation is an indispensable stabilization technology, as it isolates CLCs from external disturbances while retaining their thermochromic activity [13,14,15,16]. Nevertheless, CLC microcapsules typically exist as micron-sized powders, making them incompatible with fiber internal doping; thus, coating technology has become the dominant approach for fabricating CLC-based textiles. This approach involves immobilizing CLC microcapsules on fabric surfaces and leveraging the temperature-induced ordered arrangement transition of CLC molecules to achieve color change via light transmission and reflection [17,18,19,20].

Despite its necessity, microencapsulation inherently compromises the color-change sensitivity and color-rendering performance of CLCs—a critical limitation that has hindered their application in high-precision temperature-responsive scenarios [21,22,23]. To address this challenge, we leverage MXene, an emerging class of two-dimensional (2D) nanomaterials with distinct advantages (e.g., high electrical conductivity, large specific surface area, and abundant surface functional groups) that have been proven to enhance thermal response and interfacial compatibility in polymer composites [24,25]. Notably, the integration of MXene as a thermal response enhancer into CLC microcapsule-based textile systems to mitigate microencapsulation-induced performance degradation remains underexplored. Unlike previous studies that used MXene for conductivity enhancement or structural support, this work leverages MXene’s high thermal response and interfacial compatibility to address the long-standing bottleneck of CLC sensitivity loss post-microencapsulation. We thus propose a synergistic design: fabrics serve as the substrate, CLC microcapsules as the color-changing response unit, and MXene as the thermal response enhancement medium to construct high-sensitivity thermochromic textiles.

To implement this synergistic design, we first fabricated stable MXene nanosheet dispersions via hydrochloric acid-lithium fluoride (HCl-LiF) etching, dimethyl sulfoxide (DMSO) intercalation, and phytic acid dispersion of Ti_3_AlC_2_ powder. To resolve the electrostatic repulsion between negatively charged MXene nanosheets and cotton fabrics, the fiber substrates were pre-modified with polydopamine (PDA) to introduce positive surface charges, followed by the construction of poly(diallyldimethylammonium chloride) (PDAC)/MXene composite coatings via layer-by-layer (LbL) self-assembly; subsequent hydrophobic modification yielded PDA@PDAC/MXene functional substrates with enhanced environmental stability. Ultimately, CLC microcapsules were immobilized on the functional substrate surfaces via a coating process to obtain MXene-based thermochromic fabrics. We systematically characterized the morphology, structure, and composition of MXene nanosheets, liquid crystal microcapsules, and the resultant thermochromic fabrics, while investigating the influence of MXene loading on the thermochromic response performance. This work is dedicated to developing flexible, high-sensitivity thermochromic liquid crystal fabrics, expanding the non-display application scope of CLCs, advancing the fabrication technology of thermochromic textiles, and laying a technical foundation for meeting the diverse demands of the smart wearable market.

## 2. Results and Discussion

### 2.1. Preparation and Characterization of MXene

**Figure 1:**
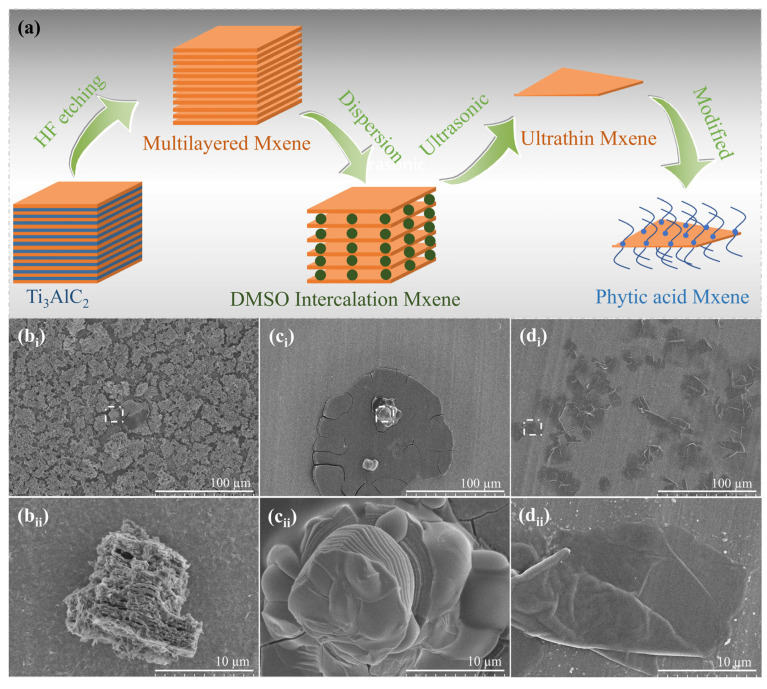
Preparation process of MXene and scanning electron microscopy (SEM) images of products at different preparation stages (**a**) is the preparation process route of MXene, and a stable dispersion of MXene nanosheets is obtained through a three-step method of etching, intercalation-exfoliation, and dispersion; (**b_i_**,**b_ii_**) are SEM images of pristine Ti_3_AlC_2_ powder; (**c_i_**,**c_ii_**) are SEM images of MXene after DMSO intercalation; (**d_i_**,**d_ii_**) are SEM images of MXene nanosheets dispersed with phytic acid.

MXene nanosheets act as the core thermal response enhancer in this work, and their successful preparation and structural characterization are crucial for the subsequent fabrication of composite fabrics. The preparation process of MXene nanosheets is illustrated in Figure 1a, with a stable dispersion obtained via three sequential steps: etching, intercalation-exfoliation, and dispersion [26,27,28]. Initially, LiF and HCl were employed as composite etchants to treat Ti_3_AlC_2_, yielding Ti_3_C_2_T_x_ (T_x_ denotes surface functional groups, e.g., -OH, -O, -F). Subsequently, positively charged small molecules (e.g., Li^+^, DMSO) were intercalated between the negatively charged Ti_3_C_2_T_x_ layers via electrostatic attraction to attenuate interlayer forces. Monolayer or few-layer MXene solutions were then achieved by mechanical external forces, including mechanical vibration or water bath ultrasound. Ultimately, phytic acid was utilized to disperse the monolayer and few-layer MXene, resulting in a stable dispersion of MXene nanosheets.

To elucidate the morphological evolution of Ti_3_C_2_T_x_ etched by HCl-LiF, scanning electron microscopy (SEM) was utilized to characterize the reactants, intermediates, and final products during the entire preparation process. SEM micrographs of the reactants are presented in Figure 1(b_i_,b_ii_), revealing that Ti_3_AlC_2_ possesses a layered close-packed morphology with narrow gaps and a rough surface. As illustrated in Figure 1(c_i_,c_ii_) (post-DMSO intercalation), MXene exhibits expanded interlayer spacing, a smooth surface, and a well-defined layered structure following etching and intercalation. This phenomenon is due to the spontaneous insertion of metal ions (Li^+^) and water molecules between the stacked MXene layers during the preparation process, which weakens the interlayer forces and thus increases the interlayer spacing. Subsequently, through intercalation and mechanical external forces, single-layer or few-layer MXene nanosheets were generated, and the results are shown in Figure 1(d_i_,d_ii_).

**Figure 2:**
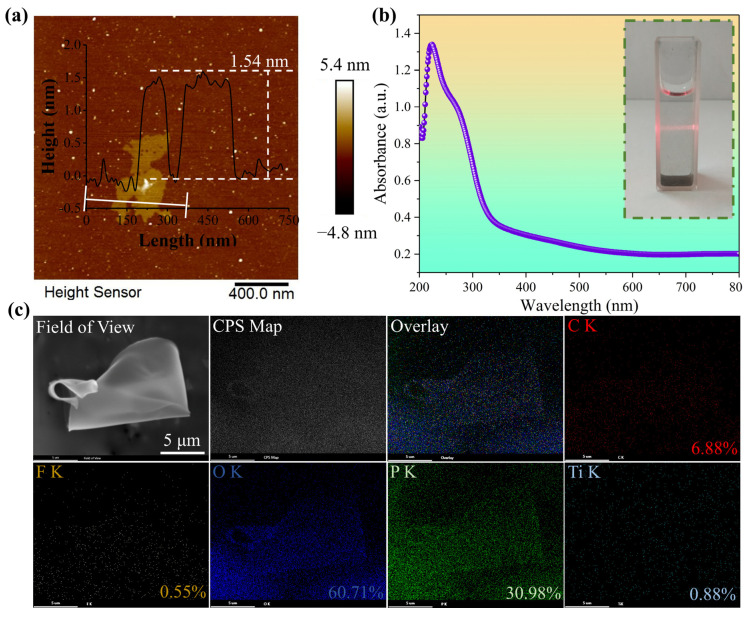
(**a**) Atomic force microscopy (AFM) image; (**b**) Ultraviolet-visible absorption spectrum and Tyndall effect physical image; (**c**) Scanning electron microscopy element mapping (SEM mapping) image of MXene.

To further explore the microstructure of MXene nanosheets, AFM and SEM were used to characterize their thickness and elemental composition in detail. According to the results in Figure 2, MXene nanosheets feature a thin-layered structure with an average thickness of 1.54 nm, which is consistent with the reported thickness of monolayer/few-layer MXene [29,30,31]. From the elemental distribution map under SEM in Figure 2c, it can be seen that C, O, P, Ti, and a small amount of F elements are uniformly distributed on the MXene nanosheets. Therefore, based on the morphology and size, it can be concluded that nanoscale two-dimensional MXene nanosheets have been successfully prepared. In the ultraviolet absorption spectrum of Figure 2b, the absorption peak of MXene nanosheets is approximately at 250 nm, and from the inset in the upper right corner, it can be seen that when a red laser pointer is irradiated into the MXene dispersion, a bright light path is observed, indicating that the solution is uniformly dispersed, has the Tyndall effect, and possesses colloidal characteristics.

### 2.2. Preparation and Characterization of Cholesteric Liquid Crystal Microcapsules

**Figure 3:**
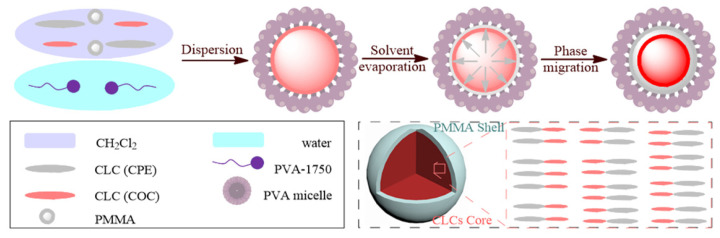
Schematic presentation of cholesteric liquid crystal microcapsules.

Cholesteric liquid crystal microcapsules (CLCMs) were prepared by the solvent evaporation method, and their preparation process is shown in Figure 3. The preparation process mainly consists of two core steps: first, the polyvinyl alcohol (PVA) aqueous solution is stirred together with the mixed liquid crystals dissolved in dichloromethane; second, the evaporation rate of dichloromethane and PVA is controlled by heating to obtain CLCMs.

**Figure 4:**
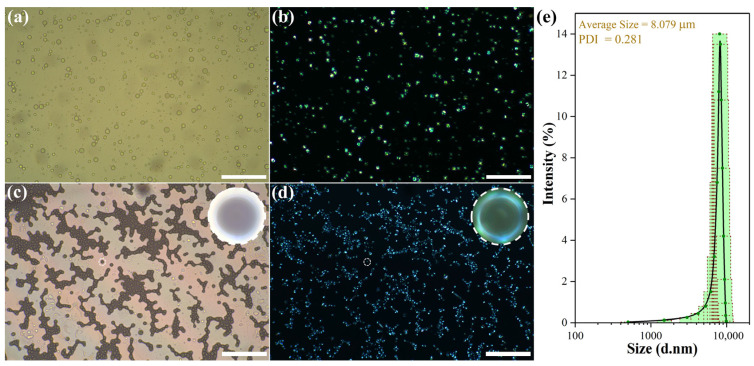
(**a**) Optical microscopy (OM) and (**b**) polarized optical microscopy (POM) images of microcapsule emulsion; (**c**) OM and (**d**) POM images of dried microcapsules (scale bar: 200 μm); (**e**) Particle size distribution (PSD) histogram of CLCMs (inset: mean value and standard deviation).

Figure 4 presents morphological images of the emulsion and dried microcapsules under normal light (optical microscopy, OM) and polarized light (polarized optical microscopy, POM). Figure 4a clearly shows that the microcapsules exhibit a spherical structure, stable dispersion in the solution, low tendency for agglomeration, and relatively uniform particle size. After drying, the microcapsules retain a spherical shape (Figure 4c) without demulsification, demonstrating excellent structural stability. In addition, under polarized light conditions (see Figure 4b,d), the microcapsules show obvious cross-shaped extinction patterns. This is because poly(methyl methacrylate) (PMMA) does not show color under polarized light, while the supramolecular spatial structure of liquid crystals exhibits a regular polarized light effect, indicating that the liquid crystals are successfully encapsulated inside the microcapsules [32,33]. The PSD histogram (laser particle size analyzer) shows a normal distribution with a mean particle size of 8 μm and a PDI of 0.28 (Figure 4e), which quantitatively verifies uniform particle size. Thus, it can be inferred that CLCMs with uniform particle size distribution have been successfully prepared.

### 2.3. Characterization and Color-Changing Performance of MXene-CLCs-Based Fabrics

Layer-by-layer (LbL) self-assembly is an effective method for preparing MXene-functionalized fabrics [34,35]. However, cotton fabrics are negatively charged in aqueous solutions, and MXene nanosheets dispersed with phytic acid are also negatively charged, making it difficult to introduce MXene onto the fabric surface through self-assembly. In this experiment, PDA was first used for cationic pretreatment of pristine cotton fabrics to make the fabric surface positively charged, and then the negatively charged phytic acid-dispersed MXene nanosheets were successfully adsorbed onto the fibers through electrostatic attraction. In addition, the number of cycles of PDA modification and phytic acid-dispersed MXene nanosheet adsorption can effectively control the number of assembled layers of MXene nanosheets on the fabric surface.

**Figure 5:**
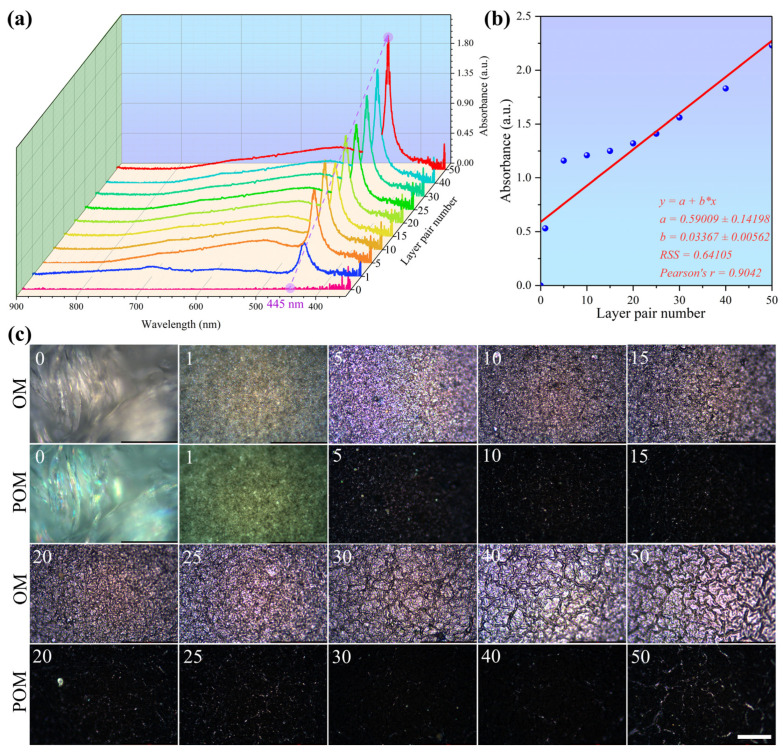
(**a**) Relationship between the number of assembly cycles and the absorption spectrum curves of PDA@PDAC/MXene fabrics; (**b**) Relationship between the number of assembly cycles and the absorbance at the maximum absorption wavelength of PDA@PDAC/MXene fabrics; (**c**) OM and POM images of PDA@PDAC/MXene with different assembly cycles (scale bar: 200 μm).

A UV-Vis spectrometer and a microscope were used to characterize the PDA@PDAC/MXene fabrics, and the response relationship between the number of assembly cycles and the performance of PDA@PDAC/MXene fabrics was analyzed. As shown in Figure 5a, the maximum absorption wavelength of PDA@PDAC/MXene fabrics is 445 nm, indicating that increasing the number of assembly cycles does not affect the absorption wavelength of the characteristic peak of the spectral curve and thus does not affect its intrinsic properties [36]. When the number of assembly cycles is 0, 1, 5, 10, 15, 20, 25, 30, 40, and 50, with the continuous increase in the number of assembly cycles, the absorption peak of the ultraviolet absorbance of PDA@PDAC/MXene fabrics shows a gradual increasing trend (Figure 5b), indicating that the more the MXene loading, the stronger the ultraviolet absorption capacity. From the OM images of the actual samples in Figure 5c, it can be seen that with the increase in the number of assembly cycles, the gaps between molecules are continuously filled, and the MXene on the fabric surface gradually extends into a film from a scattered distribution, forming a more continuous thermal conduction network. Combined with literature-reported MXene thermal conductivity, the increased MXene loading (positively correlated with assembly cycles, Figure 5b) enhances the network’s integrity: scattered MXene nanosheets (low loading) result in discontinuous heat transfer, while film-forming MXene (high loading) enables efficient heat conduction across the fabric surface.

**Figure 6:**
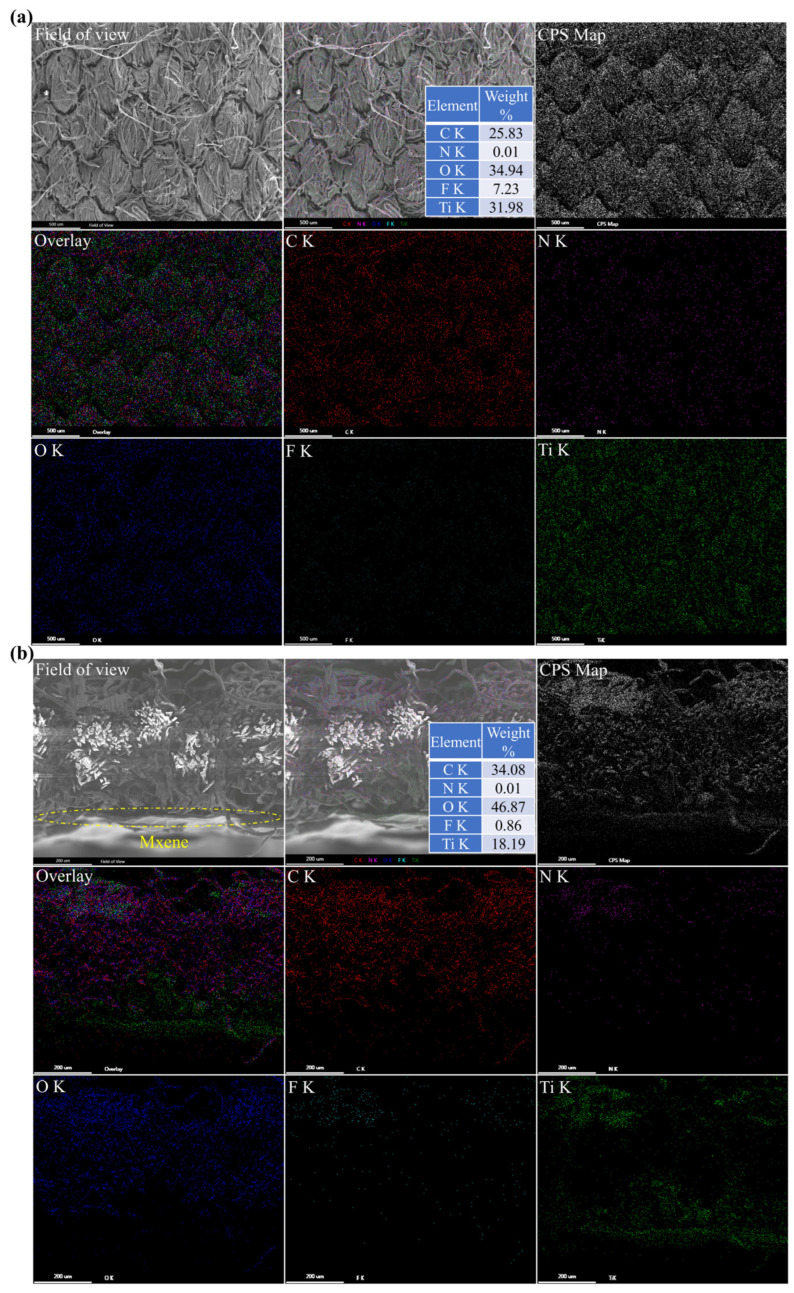
SEM-Mapping images of (**a**) surface and (**b**) cross-section of PDA@PDAC/MXene fabrics.

From the SEM images of the surface and cross-section of PDA@PDAC/MXene fabrics in Figure 6, it can be seen that the fiber outline on the surface of PDA@PDAC/MXene fabrics is blurred, which is due to the deposition of MXene on the fiber surface and between the fiber gaps. In addition, elemental distribution maps were used to further analyze the distribution state of MXene inside the fabrics. The five main elements (C, N, O, F, Ti) are uniformly distributed on the surface and cross-section of the fabrics, and the distribution of the Ti element is almost identical to the distribution trajectory of the fibers. In addition, in the overall elemental content distribution, Ti and F elements account for a relatively large proportion, further confirming that MXene is successfully covered on the fabric surface.

**Figure 7:**
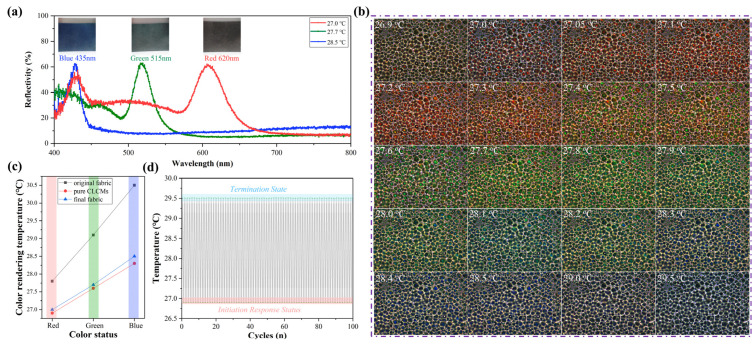
(**a**) Reflectance spectrum curves, digital photographs (2 cm × 2 cm prototype, naked-eye view) of the textile prototypes at different color states under varying temperatures (26.5–29.5 °C); (**b**) OM images of liquid crystal microcapsules in the color-changing layer of fabrics during heating; (**c**) Temperature comparison of pure microcapsules, original fabric and final fabric at different color-developing states; (**d**) Durability test of the textile’s color-changing response temperature.

Figure 7a supplements the reflectance spectrum curves and optical images of the final textile prototypes: digital photographs captured at 90° (lateral) angles, corresponding to the three characteristic colors (red at 27.0–27.5 °C, green at 27.6–28.2 °C, blue at 28.3–29.5 °C) during the thermochromic transition. The images confirm that the textile maintains uniform color distribution with no obvious color distortion, which is attributed to the homogeneous dispersion of MXene and CLCMs on the fabric surface. The reflectance spectrum curves are consistent with the visual color changes, further validating the high sensitivity of the thermochromic response. A hot-stage polarized optical microscope was used to characterize the color-changing performance of the liquid crystal microcapsules in the color-changing functional layer of the fabrics, and the results are shown in Figure 7b. The liquid crystal microcapsules exhibit a color-transition temperature range of 26.5–29.5 °C. Upon heating, the color transitions sequentially as red → orange → yellow → green → cyan → blue → purple. Among them, red is displayed at 27.0~27.5 °C, green at 27.6~28.2 °C, and blue at 28.3~29.5 °C. This is consistent with the thermochromic temperature of the pristine liquid crystals [37,38], indicating that the introduction of MXene effectively reduces the damage to the thermochromic sensitivity of liquid crystals caused by microencapsulation. Figure 7c presents a direct comparative analysis of thermal response performance among three groups: MXene-enhanced fabrics (this work), control fabrics (without MXene), and pure CLCMs, all tested under identical environmental conditions. MXene-enhanced fabrics exhibit a narrow temperature range (27.0–28.5 °C) closer to pristine CLCMs (26.9–28.3 °C) than control fabrics (27.8–30.5 °C). Control fabrics show a broader and shifted temperature range due to inefficient heat transfer—without MXene, heat accumulation on the fabric surface leads to local temperature fluctuations, causing inconsistent CLCM molecular orientation transitions. In addition, the results of Figure 7d indicate that the textile maintains stable thermochromic performance during repeated use, with no obvious fatigue.

## 3. Materials and Methods

### 3.1. Materials

PDAC [molecular weight (Mw) = 200,000 to 350,000 g/mol, 20 wt% in water], linear poly(ethyleneimine) (Mw = 50,000 g/mol), hydrochloric acid [HCl; ACS reagent; 37% (*w*/*w*)], and dimethyl sulfoxide (DMSO; Reagent Plus; >99.5%) were purchased from Sigma-Aldrich(Shanghai)Trading Co., Ltd., Shanghai, China. LiF (98+% purity) was purchased from Shanghai Aladdin Biochemical Technology Co., Ltd., Shanghai, China. Ti_3_AlC_2_ powder (200 mesh, 98%) was purchased from 11 Technology Co., Ltd., Changchun, China.

Cholesteryl-pelargonate (CPE, purity N95%) was obtained from Alfa Aesar (China) Chemical Co., Ltd., Shanghai, China. Cholesterol-oleyl-carbonate (COC, purity N95%) was supplied by Tokyo Chemical Industry Co., Ltd., Tokyo, Japan. PMMA (Mw = 5.0 × 10^4^–1.0 × 10^5^ g/mol, refractive index 1.49) was purchased from Sinopharm Chemical Reagent Co., Ltd., Shanghai, China. Polyvinyl alcohol (PVA-1750), of reagent grade without further purification, were purchased from Shanghai Macklin Biochemical Co., Ltd., Shanghai, China. The organic solvents, such as dichloromethane (CHCl_2_) and ethyl acetate of analytical grade, were purchased from Sinopharm Chemical Reagent Co., Ltd., Shanghai, China. The distilled deionized water was used as received.

Tris(hydroxymethyl)aminomethane-hydrochloride [Tris-HCl, ≥99%], dopamine hydrochloride (DPA, ≥98%), sodium hydroxide (NaOH, AR), 1H,1H,2H,2H-perfluorooctyltriethoxysilane (PFTTS, ≥97%), and ethanol absolute (EG) were purchase form Shanghai Macklin Biochemical Co., Ltd., Shanghai, China.

### 3.2. Preparation of Ti_3_C_2_T_x_

Ti_3_C_2_T_x_ was fabricated via etching and exfoliation of Ti_3_AlC_2_ powder using a hydrochloric acid-lithium fluoride (HCl-LiF) composite etching system. Initially, an etching solution was formulated by mixing 37% (*w*/*w*) concentrated HCl with deionized water to prepare 20 mL of 9 mol/L HCl aqueous solution, which was subsequently transferred to a polytetrafluoroethylene (PTFE) beaker and magnetically stirred for 5 min to achieve homogenization. Subsequently, 1.00 g Ti_3_AlC_2_ powder was gradually added under continuous stirring to prevent local agglomeration. The beaker was sealed with Parafilm (3–5 small holes were drilled to release reaction-generated gases and avoid pressure buildup) and immersed in a constant-temperature water bath at 40 °C for magnetic stirring over 48 h.

After the reaction, the resulting mixture was transferred to a centrifuge tube, deionized water was added, and centrifugation was performed at 3500 rpm for 5 min to discard the supernatant and retain the precipitate. An appropriate amount of deionized water was added to the precipitate for ultrasonic dispersion (500 W power, 40 kHz frequency) over 30 min, followed by another centrifugation at 3500 rpm for 5 min; this washing-centrifugation cycle was repeated 5~6 times until the supernatant showed a stable dark green color and a pH value of 6.8~7.2 (close to neutral). Finally, the purified swollen clay-like sediment was dispersed into 50 mL of deionized water, shaken for 3 min to form a suspension, centrifuged at 3500 rpm for 30 min, and the supernatant was collected to obtain Ti_3_C_2_T_x_ dispersion, which was stored in a refrigerator at 4 °C for later use.

### 3.3. Preparation of MXene Nanosheet Dispersion

The preparation of MXene nanosheet dispersion involved three sequential steps: DMSO intercalation, ultrasonic exfoliation, and phytic acid dispersion. Initially, an appropriate amount of DMSO and the previously prepared Ti_3_C_2_T_x_ dispersion were mixed with deionized water to form a 60 mg/mL suspension, which was stirred magnetically at room temperature for 18 h to achieve sufficient intercalation of DMSO molecules between the Ti_3_C_2_T_x_ layers. After the intercalation reaction, the suspension was transferred to a centrifuge tube and centrifuged at 5000 rpm for 2 h to discard the supernatant and retain the precipitate, with this centrifugation step repeated 2~3 times to completely remove excess DMSO. An appropriate amount of deionized water was added to the purified precipitate to adjust the concentration to 20 mg/mL, which was ultrasonically treated in a water bath (400 W power, 40 kHz frequency, ice bath cooling to avoid overheating) for 1 h and subsequently centrifuged at 3500 rpm for 1 h, with the supernatant collected as DMSO-intercalated MXene nanosheet dispersion. Finally, 3 mL of 50% (*w*/*w*) phytic acid solution was added dropwise (1 mL/min rate) to the MXene nanosheet dispersion, and ultrasonic treatment (300 W power, 40 kHz frequency) was performed for 2 h at 25 ± 2 °C to allow phytic acid molecules to graft onto the surface of MXene nanosheets through chemical bonding or electrostatic interaction, thereby enhancing their dispersion stability. The resulting stable dispersion of phytic acid-dispersed MXene nanosheets was stored in a refrigerator at 4 °C for later use.

### 3.4. Preparation of PDA-Modified Substrates

PDA was coated on the substrate surface via in situ polymerization. First, the substrate to be modified (cotton fabric, PET film, etc.) was cut into 5 cm × 5 cm samples, which were ultrasonically cleaned in isopropanol, anhydrous ethanol, and deionized water for 15 min each to remove surface oil and impurities; after cleaning, the samples were placed in a vacuum drying oven and dried hermetically at 60 °C for later use. A Tris-HCl buffer solution was then prepared by adding 156.6 mg Tris and 100 mL deionized water to a 250 mL beaker, stirring to dissolve, adjusting the pH value to 8.5 with 37% concentrated HCl, and stirring uniformly in a constant temperature water bath at 25 °C. Next, 300 mg DPA was added to the buffer solution, and magnetic stirring was conducted for 5 min until complete dissolution. The pretreated substrate was then added to the mixture, with continuous magnetic stirring at 25 °C for 24 h to facilitate the in situ oxidative polymerization of DPA on the substrate surface to form PDA. After the reaction, the substrate was taken out, excess reaction solution on the surface was gently squeezed out, and the substrate was rinsed repeatedly with deionized water 3~5 times to remove weakly adsorbed PDA particles. The cleaned substrate was placed in a vacuum drying oven and dried hermetically at 60 °C for 2 h to obtain the PDA-coated modified substrate (PDA@substrate), which was sealed and stored for later use.

### 3.5. Preparation of PDAC/MXene Electrostatic Self-Assembled Coated Fabrics

PDAC/MXene composite-coated fabrics were prepared using vacuum filtration-assisted electrostatic self-assembly technology. Firstly, the MXene nanosheet dispersion prepared in Section 3.3 was ultrasonically treated for 30 min to ensure uniform dispersion, then diluted with deionized water to a concentration of 1 mg/mL and adjusted to pH 5.0; simultaneously, a 1 mg/mL PDAC aqueous solution was prepared and adjusted to pH 5.0. A filtration device was constructed by cutting water-based filter paper to match the size of the Buchner funnel, wetting it, and attaching it closely to the filter cloth at the bottom of the funnel, followed by placing the PDA@substrate tightly on the filter paper surface to ensure no air bubbles between the substrate and the filter paper. Next, 10 mL of 1 mg/mL PDAC aqueous solution was poured into the Buchner funnel, and a vacuum filtration pump was turned on to allow PDAC molecules to be adsorbed onto the PDA@substrate surface through electrostatic attraction, achieving cationization of the substrate; after filtration, the substrate surface was rinsed with 5 mL deionized water to remove unabsorbed free PDAC. Subsequently, 10 mL of 1 mg/mL MXene dispersion was poured into the Buchner funnel, and vacuum filtration was continued to enable MXene nanosheets to be adsorbed onto the cationized substrate surface via electrostatic attraction; after filtration, the substrate was rinsed with 5 mL deionized water to remove unabsorbed MXene nanosheets. Finally, the adsorbed substrate was placed in a vacuum drying oven and dried at 60 °C for 1 h to obtain the PDA@PDAC/MXene composite-coated fabric. The MXene loading on the substrate surface was controlled by repeating the filtration adsorption-drying cycle of PDAC and MXene, with the substrate fully rinsed with deionized water after each cycle.

### 3.6. Preparation of Hydrophobically Modified PDA@PDAC/MXene Substrates

The PDA@PDAC/MXene substrates were hydrophobically modified with perfluorosilane. First, a modification solution was prepared by adding 50 mL of anhydrous ethanol, 0.125 mL deionized water, and 0.25 g 1H,1H,2H,2H-perfluorooctyltriethoxysilane to a 100 mL beaker, which was then stirred uniformly in a constant temperature water bath at 25 °C for 30 min. The PDA@PDAC/MXene substrate prepared in Section 3.5 was completely immersed in the modification solution, and magnetic stirring was maintained at 25 °C for 5 min to allow perfluorosilane molecules to undergo a dehydration condensation reaction with hydroxyl groups on the substrate surface and graft onto the surface. The modified substrate was then taken out, placed in a vacuum drying oven, and dried at 80 °C for 2 h to ensure the full progress of the grafting reaction, ultimately obtaining a PDA@PDAC/MXene substrate with hydrophobic properties, which was sealed and stored for later use.

### 3.7. Preparation of Cholesteric Liquid Crystal Microcapsules

Cholesteric liquid crystal microcapsules (CLCMs) were prepared via the solvent evaporation method. Initially, CPE and COC were mixed at a mass ratio of 1:1 and heated with stirring in a constant temperature water bath at 60 °C for 10 min to form a uniform mixed cholesteric liquid crystal. Next, 1.00 g of the mixed cholesteric liquid crystal and 0.15 g PMMA were sequentially added to a mixed solvent containing 8.5 mL DMC and 1.5 mL ethyl acetate, and magnetic stirring was performed for 30 min until complete dissolution to form a uniform oil phase solution. A water phase was prepared by weighing 0.80 g PVA, adding it to 40 mL deionized water, heating to 80 °C with stirring to dissolve, and cooling to room temperature to obtain a 2 wt% PVA aqueous solution. For emulsification, 40 mL of the 2 wt% PVA aqueous solution was added to a 100 mL three-necked flask, magnetic stirring was turned on, the rotation speed was adjusted to 1500 rpm, and the system temperature was controlled at 20 °C; the oil phase solution was added dropwise (0.5 mL/min rate) into the three-necked flask via a syringe pump within 2 min, and stirring was continued at 1500 rpm (rotor diameter 30 mm) for 20 min at 20 ± 1 °C to form a stable oil-in-water (O/W) emulsion (droplet size 100~200 μm verified by OM). The stirring speed was then reduced to 300 rpm, and after the emulsion stabilized, another 40 mL of 2 wt% PVA aqueous solution was added to the three-necked flask; the water bath temperature was raised from 20 °C to 35 °C at a rate of 0.75 °C/min within 20 min, and this temperature (35 ± 1 °C) and stirring speed (300 rpm, rotor diameter 30 mm) were maintained while the reaction system was stirred in an open environment for exactly 10 h to fully evaporate the organic solvent (residual solvent content < 0.1% verified by gas chromatography) in the oil phase and solidify the PVA wall material to form microcapsules. After the reaction, the reaction solution was transferred to a beaker, diluted with deionized water, and allowed to stand for sedimentation for 12 h to discard the supernatant; this water washing-sedimentation cycle was repeated 3 times, and the precipitate was then vacuum-dried for 4 h to obtain CLCM products, which were sealed for later use.

### 3.8. Preparation of MXene-Based Liquid Crystal Microcapsule Coated Fabrics

MXene-based liquid crystal microcapsule-coated fabrics were prepared by combining vacuum-assisted filtration with screen printing. Firstly, an appropriate amount of CLCM products was weighed, mixed with deionized water, and ultrasonically dispersed for 15 min to form a 10 wt% microcapsule slurry. The hydrophobically modified PDA@PDAC/MXene substrate prepared in Section 3.6 was fixed on a Buchner funnel, and the microcapsule slurry was uniformly coated on the substrate surface using vacuum filtration-assisted screen printing. Finally, the coated fabric was placed in a vacuum drying oven and dried at 60 °C for 2 h to ensure full combination between the microcapsules and the substrate, resulting in the high-sensitivity MXene-based liquid crystal microcapsule thermochromic fabric. The thermochromic performance test adopted temperature calibration: the hot stage was pre-calibrated using a standard thermometer to ensure a set temperature error ≤ 0.1 °C. During testing, the textile was closely attached to the hot-stage surface, and real-time color change images (OM/POM) and reflectance spectra were recorded to synchronously verify that the textile surface temperature matches the hot-stage control temperature.

## 4. Conclusions

This work establishes a scalable preparation protocol for MXene-reinforced CLC microcapsule thermochromic textiles. Systematic experiments and comprehensive characterizations demonstrate that the incorporation of MXene effectively mitigates the attenuation of color-changing performance in CLCs induced by microencapsulation. The as-prepared MXene-based liquid crystal microcapsule fabrics display a reversible red-green-blue color transition over 26.5–29.5 °C, which is well-matched with that of pristine CLCs, enabling high-sensitivity thermochromic responses. Meanwhile, the fabrics modified with perfluorosilane had good hydrophobic properties, improving their environmental adaptability in practical applications. Leveraging the synergistic effects between MXene and liquid crystal microcapsules, this work overcomes the long-standing bottleneck of inadequate color-change sensitivity in conventional liquid crystal microcapsule textiles. The prepared flexible thermochromic fabrics possess high response sensitivity, good structural stability, and hydrophobic performance, providing a new technical path for the development of smart wearable color-changing materials. In the future, the composite ratio of MXene and liquid crystal microcapsules can be further optimized to expand the color change temperature range, promoting their practical applications in fields such as medical temperature monitoring, smart clothing, and environmental temperature sensing.

## Data Availability

The original contributions presented in this study are included in the article. Further inquiries can be directed to the corresponding authors.

## Abbreviations

The following abbreviations are used in this manuscript:

CLCs	Cholesteric Liquid Crystals
CLCMs	Cholesteric Liquid Crystal Microcapsules
PDA	Polydopamine
PDAC	Poly(diallyldimethylammonium chloride)
LbL	Layer-by-Layer
SEM	Scanning Electron Microscopy
AFM	Atomic Force Microscopy
UV-Vis	Ultraviolet-Visible
POM	Polarized Optical Microscopy
IoT	Internet of Things
Tris	Tris(hydroxymethyl) aminomethane
DPA	Dopamine Hydrochloride
DMSO	Dimethyl Sulfoxide
CPE	Cholesteryl Nonanoate
COC	Cholesteryl Oleyl Carbonate
PMMA	Poly(methyl methacrylate)
DMC	Dichloromethane
PVA	Poly(vinyl alcohol)
O/W	Oil-in-Water
